# Beclin1 controls caspase-4 inflammsome activation and pyroptosis in mouse myocardial reperfusion-induced microvascular injury

**DOI:** 10.1186/s12964-021-00786-z

**Published:** 2021-11-03

**Authors:** Wenjing Sun, Hongquan Lu, Shujuan Dong, Rui Li, Yingjie Chu, Nan Wang, Yu Zhao, Yabin Zhang, Limeiting Wang, Lin Sun, Di Lu

**Affiliations:** 1 Department of Cardiology, Henan Provincial People’s Hospital, Zhengzhou, 450000 China; 2 Department of Nuclear Medicine, Third People’s Hospital of Honghe State, Honghe, 661000 China; 3 Science and Technology Achievement Incubation Center, Kunming Medical University, Chenggong District, 1168 West Chunrong Road, Yuhua Avenue, Kunming, 650500 Yunnan China; 4 Department of Cardiology, The Second Affiliated Hospital, Kunming Medical University, 374 Dianmian Road, Wuhua District, Kunming, 650101 China; 5 Department of Cardiology, Yunnan Geriatric Hospital, Kunming, 650501 China

**Keywords:** Ischemia/reperfusion, Caspase-4 inflammasome, Pyroptosis, Beclin1

## Abstract

**Background:**

Myocardial reperfusion injury is often accompanied by cell death and inflammatory reactions. Recently, pyroptosis is gradually recognized as pivotal role in cardiovascular disease. However, little is known about the regulatory role of beclin1 in the control of caspase-4 activation and pyroptosis. The present study confirmed whether beclin1 regulates caspase-4 mediated pyroptosis and thereby protects Human Cardiac microvascular endothelial cells (HCMECs) against injury.

**Methods:**

TTC and Evan's blue dye, western blot, immunofluorescence and immunohistochemistry staining were performed in wild mice and transgenic mice with overexpression of beclin 1(BECN1-Tg). CMECs were transfected with a beclin1 lentivirus. The cell cytotoxicity was analyzed by LDH-Cytotoxicity Assay Kit. The protein levels of autophagy protein (Beclin1, p62 and LC3II/LC3I) and caspase-4/GSDMD pathway were determined by western blot. Autophagic vacuoles in cells were monitored with RFP-GFP-LC3 using fluorescence microscope.

**Results:**

I/R caused caspase-4 activity and gasdermin D expression increase in vivo and in vitro. Overexpression of beclin1 in heart tissue and CMECs suppressed the caspase-4 activity and decreased the levels of gasdermin D; meanwhile beclin1 overexpression also reduced IL-1β levels, promoted autophagy (p62 expression was inhibited while LC3II expression was increased) in the heart and CMECs. Interestingly, beclin1 overexpression increased animal survival and attenuated myocardial infarct size (45 ± 6.13 *vs* 22 ± 4.37), no-reflow area (39 ± 5.22 *vs* 16 ± 2.54) post-myocardial ischemia reperfusion.

**Conclusions:**

Induction of beclin-1 signaling can be a potential therapeutic target in myocardial reperfusion-induced microvascular injury.

**Video Abstract**

**Supplementary Information:**

The online version contains supplementary material available at 10.1186/s12964-021-00786-z.

## Background

Acute myocardial infarction (AMI) is a leading cause of mortality and disability in the world [1, 2]. While timely reperfusion is the preferred treatment for salvage viable myocardium from infarction, it also contributes to ischemia–reperfusion injury (IRI) as an adverse consequence. Studies have been indicated that microvascular dysfunction is a critical mediator of IRI that may influence patient outcomes [2, 3] Microvascular obstruction (MVO), termed no-reflow, and microvascular leakage are the most common manifestation of microvascular dysfunction [3, 4]. However, mechanisms of the microvascular injury are complicated. Gerd Heusch indicated that cytokines/chemokines play a pivotal role in myocardial ischemia reperfusion [5]. Increasing study also demonstrated that cell death and inflammation play pivotal roles in the pathophysiology of microvascular injury following myocardial I/R [6], but its potential mechanisms have not been fully elucidated.

Several forms of cells death have been described, including apoptosis, necroptosis and pyroptosis, and they display different cellular and molecular processes and distinct outcomes. Recently, roles for pyroptosis in cardiovascular disease are emerging, including myocardial infarction, ischemia–reperfusion, diabetic cardiomyopathy and atherosclerosis [7–9]. Pyroptosis, an inflammatory programmed cell death mediated by gasdermin D (GSDMD), is triggered by cytosolic sensing of invasive infection and danger signals. It is activated by a cleavage which is mediated by caspase-1 and caspase-4/5/11, thereby releasing amino-terminal gasdermin-N and carboxy-terminal gasdermin-C domains. N-terminal fragment of GSDMD translocate to the plasma membrane, where it binds with phospholipids to form pores in membranes, leading to lytic cell death [10, 11]. Continuous activation of inflammasomes leads to excessive maturation of GSDMD, membrane pore formation and, ultimately pyroptosis. Evidence from our recent studies have indicated that caspase-1 mediates pyroptosis, which occurs during microvascular dysfunction following I/R. However, caspase-4 mediates pyroptosis and IL-1β secretion and the critical molecular mechanisms in microvascular dysfunction are largely unknown.

Autophagy influences a diverse of cellular responses, such as cell fate and inflammation [12, 13]. Beclin1, an essential protein for autophagy, have been documented to act as a contact point between autophagy and cell death[14]. But the precise mechanisms of beclin1-dependment autophagy in regulating inflammation and cell death remain elusive. It has been indicated that promoting autophagy decreases the apoptosis rate and protects the heart from the MI/R injury [15]; Yu et al. suggested that inhibition of autophagy could augment pyroptosis in doxorubicin-treated human melanoma cells [16]. Recently, it has been reported that caspase-1-medicated pyroptosis is linked to autophagy [17]. However, the underlying interaction between beclin1-dependmemt autophagy and pyroptosis in CMECs and whether autophagy can affect caspase-4-mediated pyroptosis have not been elucidated.

In this study, we report a pathway by which beclin1-driven autophagy regulates caspase-4 mediated pyroptosis and presents a new potential molecular biomarker and therapeutic target for the potential treatment of myocardial reperfusion-induced microvascular injury.

## Materials and methods

### Ischemia–reperfusion model

Adult male Wild-type (WT) and BECN1-Tg C57BL/6 J mice (18–22 g) used in the study were purchased from Cyagen Biosciences and housed under specific pathogen-free conditions. The mice were anesthetized with 0.1 ml 1% pentobarbital injection Sodium by intraperitoneal. After anesthesia, needle electrodes were inserted subcutaneously in the limbs of the mice and the standard ECG leads were recorded. The ventilator was connected after tracheal intubation with 0.12 mL tidal volume and 120–130 times/min respiratory rate. Subsequently, the hearts were then exposed between the fourth and fifth ribs and the left anterior descending (LAD) coronary artery was ligated 6–0 silk suture. It was released for 1 h, 6 h and 12 h after occlusion for 45 min. In sham-operated animals, a silk suture was passed under LAD without ligation.

### Assessment of myocardial infarct size

At 6 h after reperfusion, the region of infarct size was measured by double staining with TTC and Evan's blue dye which proceeded as previously described [18]. The area at risk (AAR) portion of the LV was stained red and white, whereas the infarct size (IS) was stained white and normal myocardium stained dark blue. The heart slices were photographed digitally. After this procedure, the images were analyzed using Image J, the IS and AAR were calculated as a percentage of the LV.

### Measurement of no-reflow area

Effect of Beclin1 on no-reflow was observed using the dual-staining as previously described [19]. In brief, at the end of I/R (45 min/6 h), the ligation suture was re-ligatured at the same occlusion location of the LAD. Subsequently, 4%, 0.1 ml thioflavin-S was injected via the inferior vena cava immediately followed by re-ligation of the LAD. Then, 3%, 0.1 ml Evan’s blue was injected via tail vein to stain the non-ischemic myocardium. The heart was harvested and washed to remove excessive Evans blue dye. The heart was isolated, froze on -80 °C refrigerator and cut into 5–6 slices. The heart slices were exposed to UV light (363 nm) using a UV transilluminator for digital photograph. Areas at risk (AAR, absence of Evans blue staining) and areas with no-reflow (attenuated thioflavin-S fluorescence) were calculated using Image J. No-reflow areas were expressed as percentage of LV.

### Measurement of microvascular leakage

Microvascular leakage was determined by Evan’s blue extravasation as previously described [20]. Briefly, Evan’s blue (1 ml, 1%) was injected via tail vein at the begin of reperfusion after ischemia. After reperfusion for 30 min, cardiac coronary network was flushed with ice-cold 1% PFA in 0.05 M citrate buffer (pH 3.5) via the LV until the perfusion solution was clear from the right atrium. The hearts were then isolated and photographed. For quantification of extravasated Evan’s blue, hearts were dissolved in formamide (400 μl/mg) on a shaker at 60 °C for 24 h. After centrifugation, the supernatants were read photometrically for the absorbance of Evan’s blue color at 610 nm with a Synergy HT plate reader (Bio-Tek, USA). Evan’s blue content was expressed as absorbance unites/gram ventricular tissue.

### Immunofluorescence and immunohistochemistry

Briefly, fresh heart, spleen and liver tissues were fixed in 4% paraformaldehyde. After this, tissues were dehydrated with a graded ethanol series and embedded in paraffin. The sections were subsequently incubated through 0.3% hydrogen peroxide in PBS to block endogenous peroxidase activity. They were then treated with 10 mM citrate buffer (pH 6.0) to retrieve the antigen, followed by rinsing in phosphate buffered saline (PBS). Thereafter, the sections were blocked with 5% goat serum in PBS for 2 h, and incubated overnight at 4 °C with the following primary antibodies: rabbit polyclonal anti-GSDMD antibody (1:100, Cell Signaling Technology), anti-F4/80 (1:200, Cell Signaling Technology). After three washes with PBS, tissues were incubated with secondary antibodies for an additional 2 h at room temperature. Captured images were further analyzed by software Image J (version 1.37; National Institutes of Health, Bethesda, MD, USA). For cytofluorescense staining and immunohistochemistry were proceeded as previously described [18].

### Cells culture and hypoxia/reoxygenation

Human cardiac microvascular endothelial cells line (HCMECs) were purchased from the Bei Na Chuanglian Biotechnology (BNCC, Wuhan, China). The passage number that we used for this study was the second or third. Cells were cultured in high glucose Dulbecco’s modified Eagle’s medium (DMEM: Hyclone, Logan, UT, USA) supplemented with 10% fetal bovine serum (BI: Israel, Middle East) and 100 IU/ml penicilin and 100 IU/ml streptomycin (Hyclone, Logan, UT, USA). The cells were cultured in a humidified environment composed of 95% air and 5% CO_2_ at 37℃. Briefly, oxygen-serum deprivation injury was occurred by placing cells in a hypoxic mosphere (1% O_2_, 5% CO_2_, 94% N_2_) in the absence of serum medium for 2 h. After 2 h, the medium was exchanged for oxygenated and serum medium, and the culture was incubated at 37 °C for 2 h.

### Lentivirus transfection

CMECs were cultured in six-well plate, On the following day, the medium was replaced with new medium containing lentivirus carrying beclin1 gene (Len-Becn1) and lentiviral vector carrying green fluorescence (Len-GFP), which were, respectively, stably transfected into CMECs for 8 h; puromycin (Thermo Scientific, A1113802) was added for further selection. Two days after transfection, H/R model of CMECs was established as described above.

### Autophagic flux measurement

Autophagic flux was monitored by transfection with RFP-GFP-LC3. CMECs were cultured in six-well plate for 24 h and subsequently transfected with RFP-GFP-LC3 lentivirus for 8–10 h. After culturing for 24–48 h, cells were fixed with 4% paraformaldehyde for 15 min. Finally, the cell nuclei were stained with DAPI. Fluorescence images were captured using a Confocal (Olympus, Tokyo, Japan) for detection of autophagosomes (yellow puncta in fusion images) and autolysosomes (red puncta in fusion images).

### Western blot analysis

Total protein was extracted from CMECs and heart, spleen and liver tissues. Protein concentration was examined by the BSA protein assay (Thermo Fisher, USA). Cell and tissue lysates were separated by SDS–PAGE using a Tris–Glycin system, and the protein bands then transferred to a PVDF membrane. The membrane was blocked with 5% fat-free milk in Tris buffered saline (TBS) for 2 h at room temperature and subsequently incubated overnight at 4℃ with the following primary antibodies: caspase-4 (1:1000, Abclonal), GDMDM (1:1000, Cell Signaling Technology), IL-1β (1:1000, Santa Cruz biotechnology), beclin1 (1:2000, Abclonal), LC3 (1:2000, Cell Signaling Technology), p62 (1:10,000, Abcam) and β-actin (1:2000, Santa Cruz biotechnology). After washing three times with TBST, membranes were incubated with horseradish peroxidase (HRP)-conjugated IgG (goat anti-rabbit secondary antibody and goat anti-mouse antibody) secondary antibodies for 2 h at room temperature. Proteins were visualized by ECL procedure (Amersham Imager 600). The expression of target proteins was standardized by β-actin.

### LDH and myocardial enzymatic assay

LDH activity in cell supernatants was evaluated using LDH-Cytotoxicity Assay Kit (Beyotime, C0017). Briefly, CMECs were plated in 96-well plates before exposure to H/R (2/2 h), H/R + 3-methyladenine (3-MA) or H/R + VX-765. LHD activity was measured at 490 nm by a microplate reader following the manufacturer’s instructions. The serum levels of creatine kinase (CK), lactate dehydrogenase (LDH) were measured using automatic biochemical analyzer (cobas c 311, Mannheim, Germany).

### Statistical analysis

Data are presented as mean ± SD and were analyzed using GraphPad Prism 6. Differences between groups were assessed by *student’s t*-tests (one measured variable) or by a two-way ANOVA with Bonferroni post hoc testing. *P* < 0.05 was considered statistically significant. Survival data from the in vivo experiments were analyzed by a log- rank test performed on curves generated by GraphPad Prism 6.0 [21].

## Results

### Ethical approval

Animal experiments were reviewed and approved by the Animal Research Ethics Committee of the Kunming Medical University.

### Becn1 overexpression attenuates myocardial reperfusion injury in mice

**Fig. 1:**
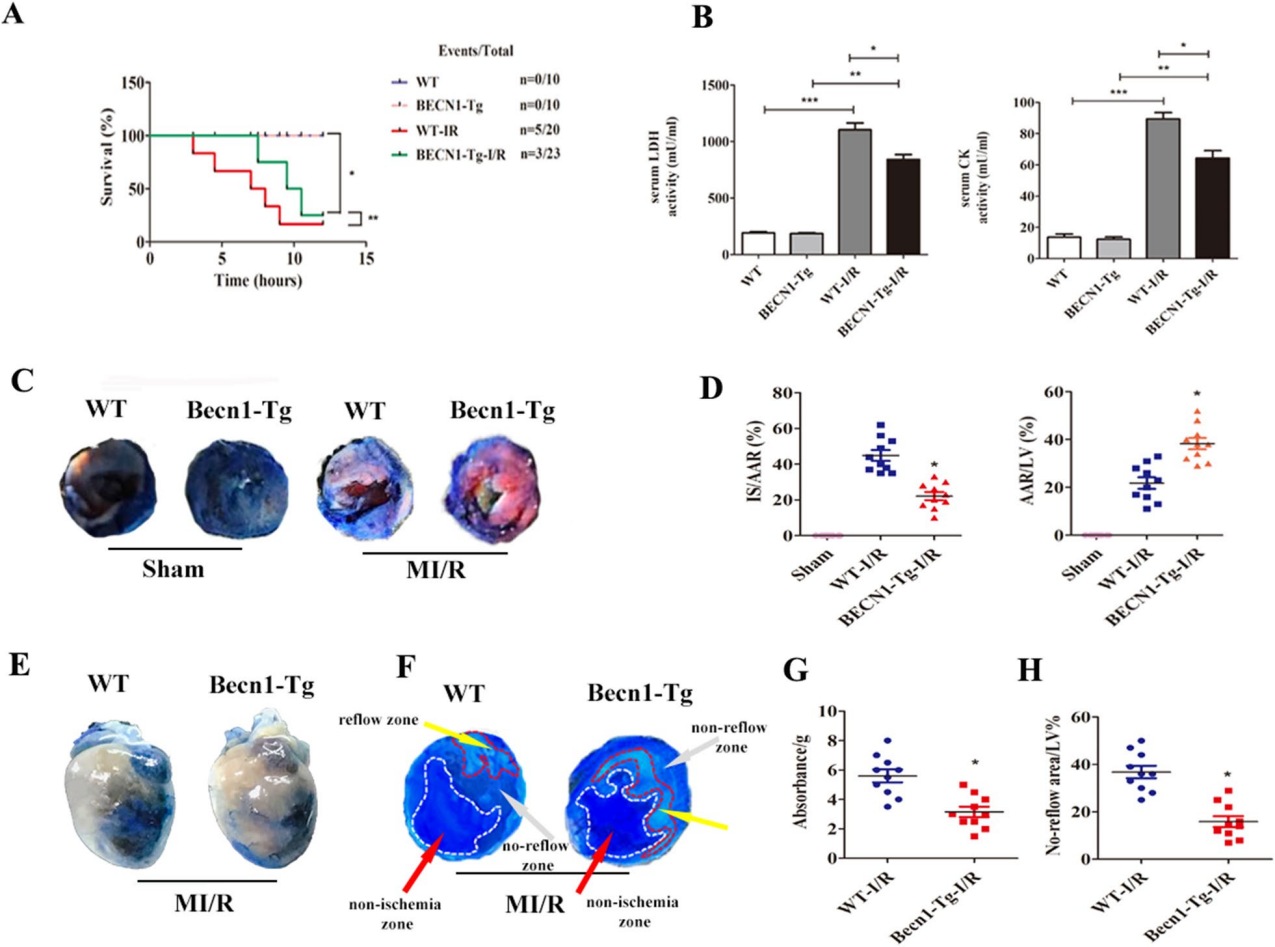
Beclin1 overexpression alleviates myocardial reperfusion injury in mice. **A** Survival analysis for mice subjected to 45 min ischemia and following 6 h perfusion (^*^P < 0.05 vs. Sham group, ^**^ P < 0.05 vs. WT-I/R group, log-rank test for survival analysis). **B** Serum concentrations of LDH and CK in each group were measured using automatic biochemical analyzer. Data are expressed as mean ± SD (n = 5 for each). ^*^P < 0.05 vs. WT-I/R group, ^**^P < 0.05 vs. BECN1-Tg group, ^***^P < 0.05 vs. WT group. **C** Representative TTC-Evan's Blue stained sections of heart from each group. Brick red-stained area represents the area at risk (AAR), whereas the white area indicates the infarcted size (IS). **D** Ratio of IS/LV, AAR/LV. Data are expressed as mean ± SD (n = 10 for each). ^*^P < 0.05 vs. WT group, ^**^P < 0.05 vs. WT-I/R group. **E** Effect of Becn1-Tg on no-reflow size and microvascular leakage.** F** Representative cross-sectional images of the left ventricular (LV) showing no-reflow regions indicated by the absence of thioflavin-S fluorescence within the ischemic area that consists of both no-reflow (white arrows) and reflow zones (yellow arrows) following IR. **G** Data are expressed as absorbent units per gram LV (absorbance/g) (n = 10 for each). ^*^P < 0.05 vs. WT-I/R group. **H** Ratio of no reflow area/LV. Data are expressed as mean ± SD (n = 10 for each). ^*^P < 0.05 vs. WT-I/R group

**Fig. 2:**
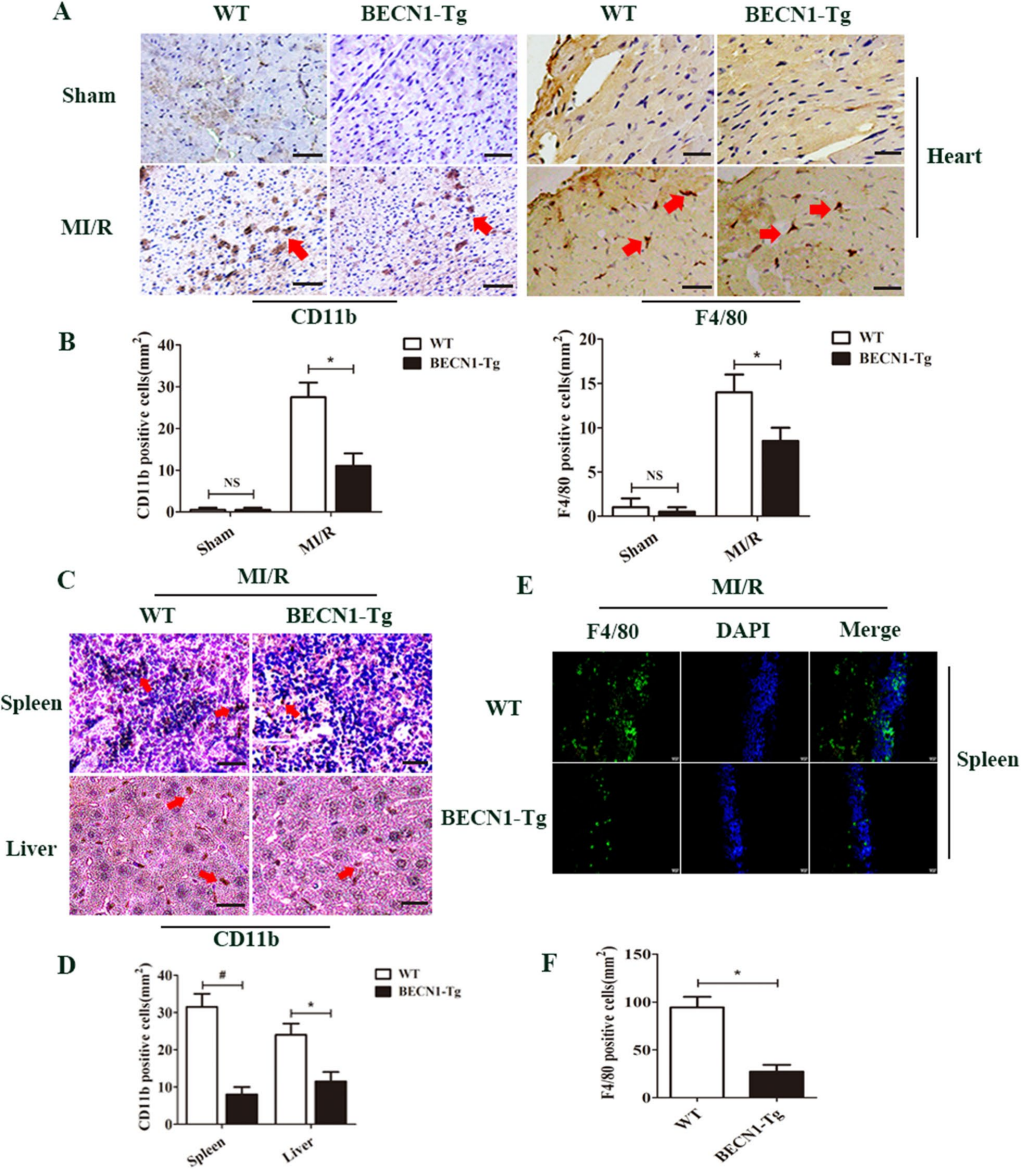
Effect of beclin1 on inflammatory cells infiltration in mice. **A** F4/80^+^ and CD11b ^+^cells were down-regulated in BECN1-Tg mice heart at 6 h after MI/R. **B** The number of F4/80^+^ and CD11b^+^ cells were quantified. **C** BECN1-Tg reduces neutrophil infiltration in spleen and liver after MI/R. **D** The percentage of CD11b^+^cells were counted in spleen and liver. **E** F4/80^+^ cells were down-regulated in BECN1-Tg mice spleen after MI/R. **F** The number of F4/80^+^ cells were quantified

Becn1 overexpression increased animal survival and decreased the levels of serum LDH and CK (Fig. 1A, B, ** < 0.05) compared to WT-I/R group. Figure 1C showed the effect of BECN1-Tg on the myocardial infarct size at 6 h after reperfusion. The ratio of myocardial infarct size to area at risk (AAR) in the MI/R group was larger than that of in the Sham group (Fig. 1D, ** < 0.05). However, the ratio of infarct size to AAR in BECN1-Tg-I/R group was smaller than that of the WT-I/R group (Fig. 1D, ** < 0.05). The AAR as the percentage of the LV (AAR/LV) in the BECN1-Tg- I/R group was significantly decreased in comparison with WT-I/R group (Fig. 1D, ** < 0.05). The microvascular damage was evaluated by microvascular permeability and no-reflow phenomenal. I/R induced microvascular permeability was attenuated in Becn1-Tg-I/R mice compared to WT-I/R mice (Fig. 1E, G, ** < 0.05). No-reflow area induced by I/R was significantly diminished in Becn1-Tg-I/R group compared to WT-I/R group (Fig. 1F, H, ** < 0.05). Our data also revealed that BECN1-Tg attenuated F4/80^+^ macrophages and CD11b^+^ neutrophils infiltration in the heart, spleen and liver compared to that of the WT group after MI/R (Fig. 2A–F, ** < 0.05).

### Becn1 overexpression enhances autophagic flux in CMECs

**Fig. 3:**
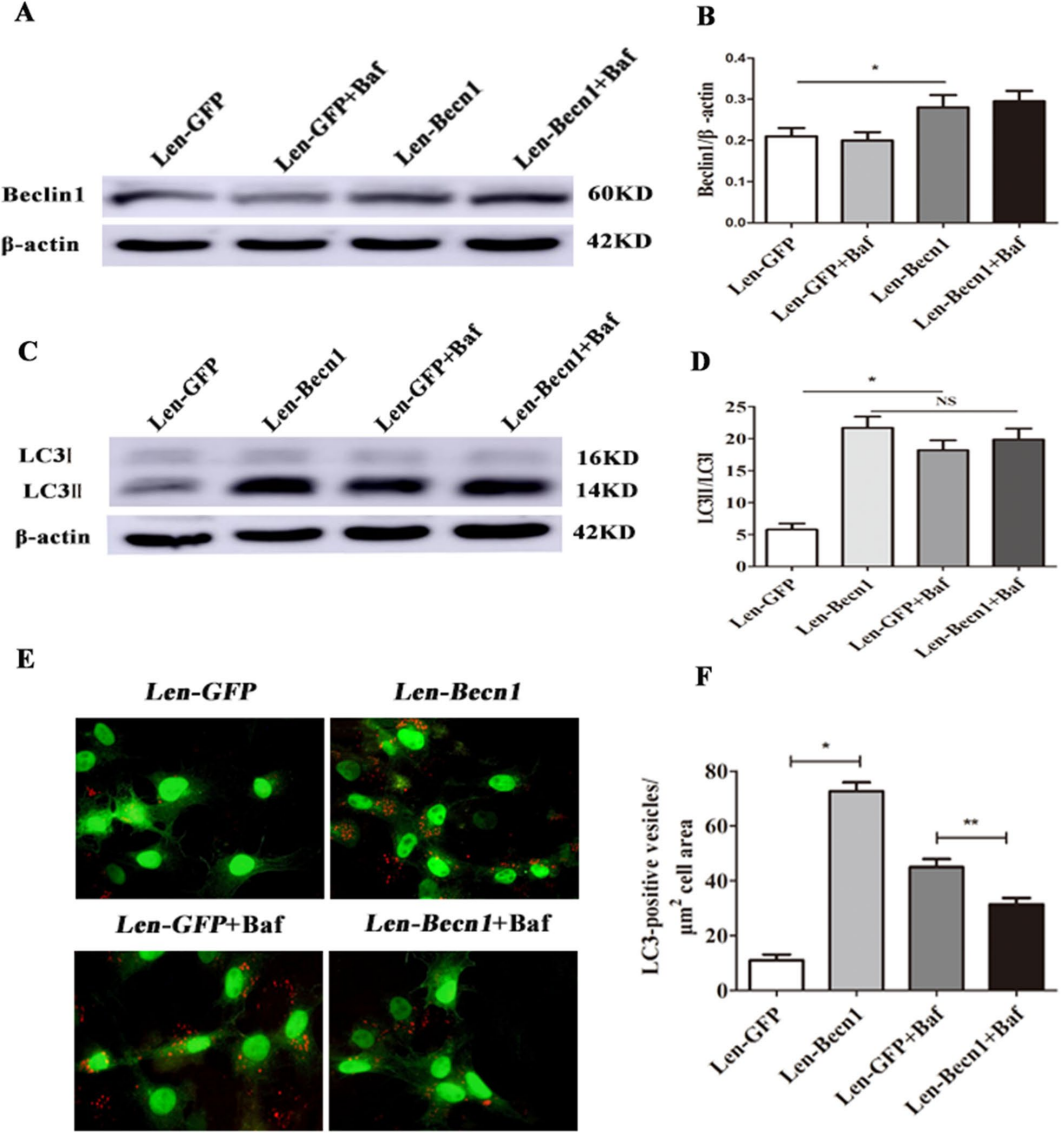
Beclin1 overexpression regulates autophagic flux in CMECs. **A**–**D** Beclin-1 and LC3 expression in Len-GFP and Len-Becn1 CMECs treated with or without Bafilomycin A1 (Baf, 50 nM) was quantified by western blotting. Data are expressed as mean ± SD (n = 3). ^*^P < 0.05 vs. Len-GFP group. **E** Endogenous LC3 expression in Len-GFP and Len-Becn1 CMECs treated with or without Bafilomycin A1 (50 nM). **F** Quantification of LC3-positive vesicles in CMECs. Data are expressed as mean ± SD (n = 3). ^*^P < 0.05 vs. Len-GFP group, ^**^P < 0.05 vs. Len-GFP + Baf group

The Becn1 content of CMECs transfected with Len-GFP and Len-Becn1 was evaluated by western blotting: The results showed Becn1 up-regulation in Len-Becn1 group (Fig. 3A, B, ** < 0.05). Following this, the effect of Becn1 overexpression on autophagy flux was determined. CMECs were cultured for 2 h in the absence or presence of Bafilomycin A1 (Baf). The results indicated that Becn1 overexpression turnover the process of Baf inhibiting autophagy flux (Fig. 3A-D). Staining of endogenous LC3-positive vesicles in Baf-treated CMECs supports these data (Fig. 3E, F).

### I/R induces caspase-4 inflammasome activation and pyroptosis in vivo and in vitro

**Fig. 4:**
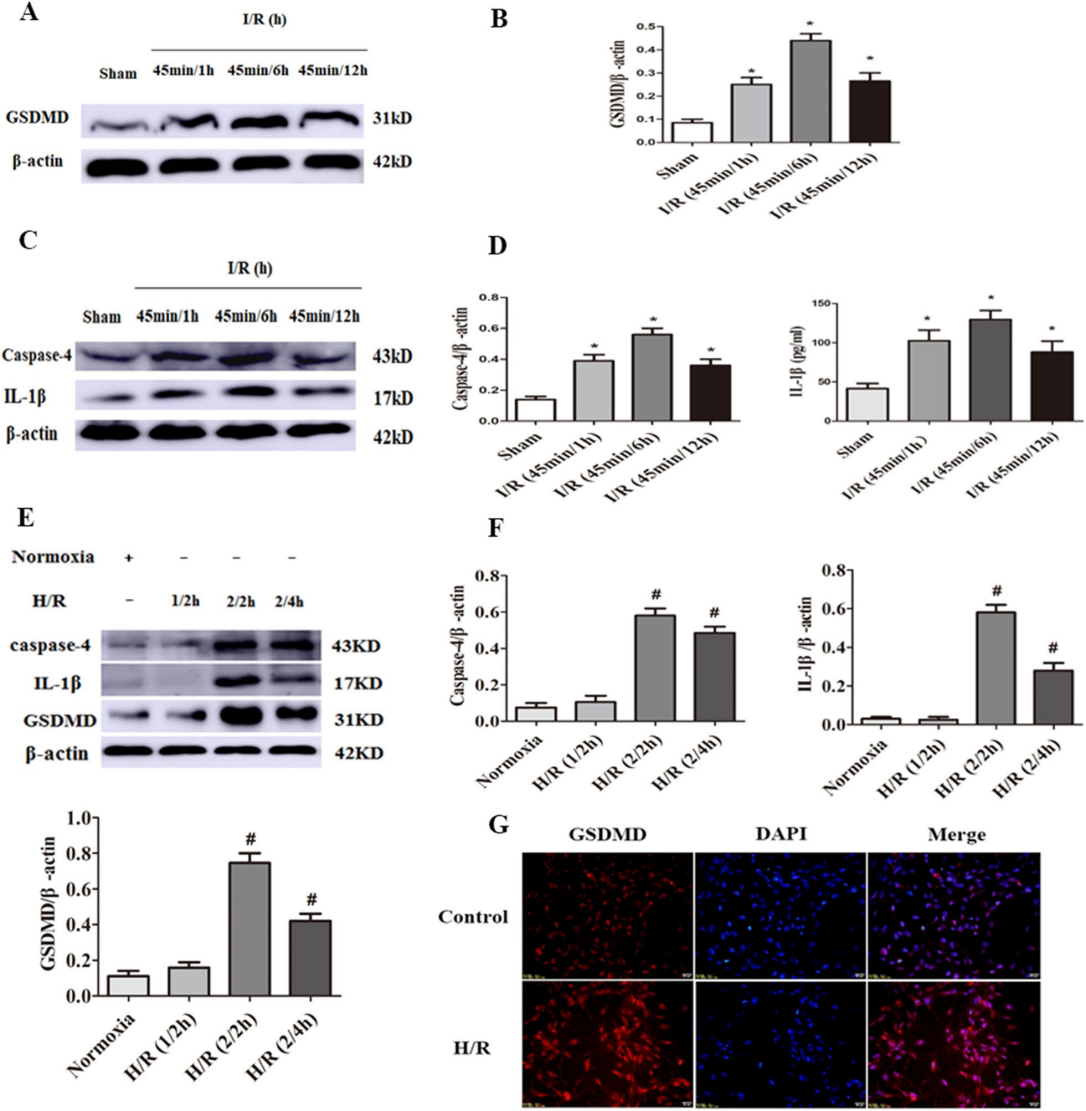
Caspase-4 inflammasome and pyroptosis-associated proteins are activated in heart and CMECs after MI/R. **A**–**D** Representative western blotting bands and densitometric quantification of GSDMD, caspase-4 and IL-1β in heart at different myocardial ischemia/reperfusion (MI/R) time points. Data are expressed as mean ± SD (n = 3). ^*^P < 0.05 vs. Sham group. **E**, **F** Representative western blotting bands and densitometric quantification of caspase-4, IL-1β and GSDMD were assessed in CMECs at different hypoxia/reoxygenation (H/R) time points. Data are expressed as mean ± SD (n = 3). ^#^*P* < 0.05 vs. control group. **G** Immunofluorescence staining for GSDMD in CMECs at 2 h of hypoxia/2 h of re-oxygenation. Scar bar = 20 μm

Studies have reported the time-dependent activation of the NLRP3 inflammasome in the heart after MI/R [22]. However, recent studies have indicated that caspase-4 is a specific molecular cell marker to trigger cell pyroptosis [23]. But the role of caspase-4-dependent pyroptosis in MI/R was unclear. Therefore, we examined the levels of caspase-4, IL-1β, GSDMD in the heart and CMECs at different reperfusion time points. The results showed that I/R induced caspase-4 activation and IL-1β secretion and resulted in higher levels of CMECs or heart pyroptosis, as determined by high expression of GSDMD in vivo and in vitro, especially at after 45 min ischemia/6 h reperfusion in vivo and 2 h of hypoxia/2 h of reoxygenation in vitro significantly increased (Fig. 4A-G, ** < 0.05). Therefore, I/R (45 min/6 h) or H/R(2/2 h)was used in subsequent experiments.

### Becn1 overexpression suppresses caspase-4 inflammasome activation and pyroptosis by enhancing autophagic flux in mice

**Fig. 5:**
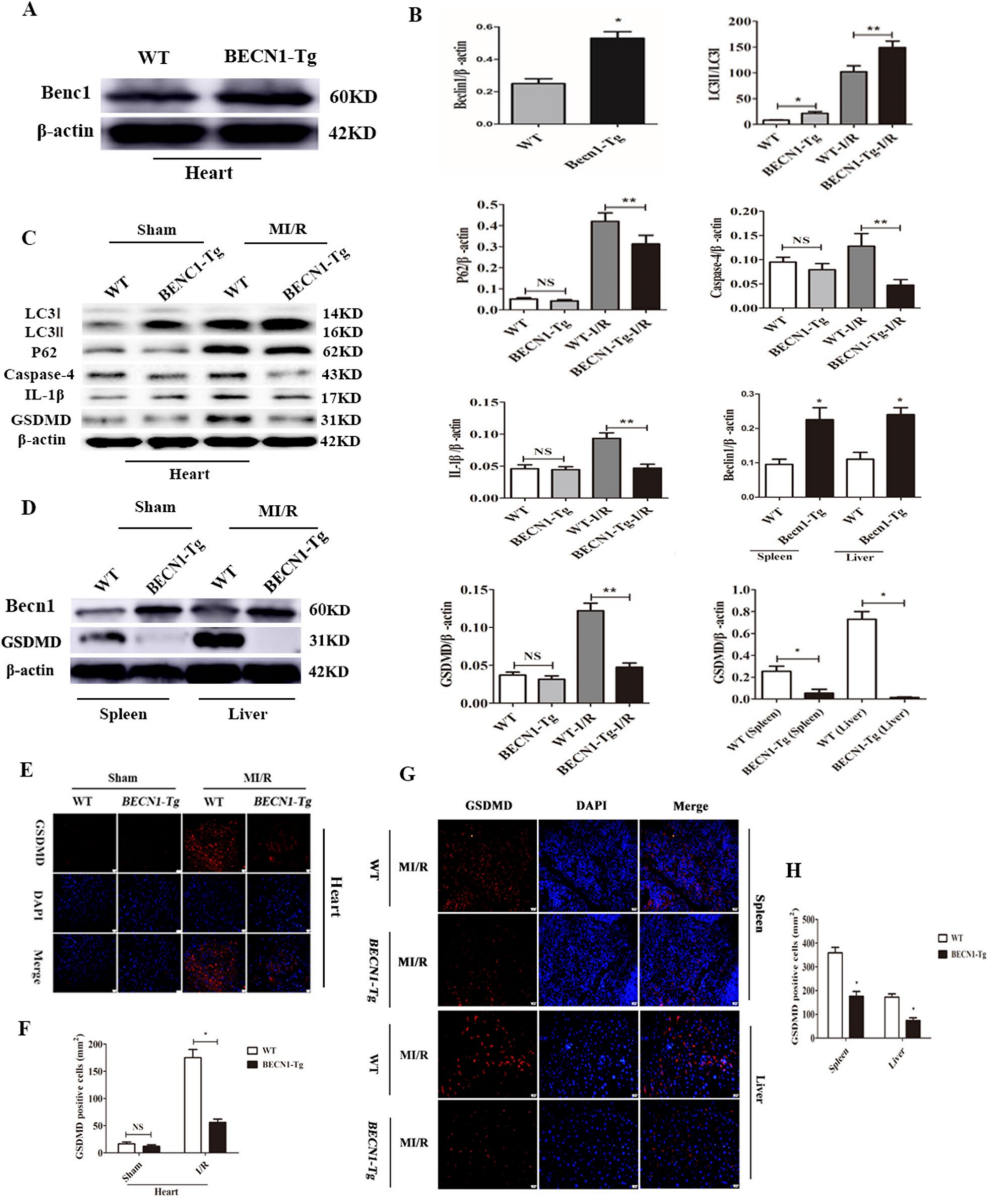
Enhanced beclin1 expression results in inhibition of caspase-4 activation and pyroptosis in mice. **A** Becn1 overexpression effect was observed. GSDMD expression in WT and BECN1-Tg heart was assessed by western blot analysis. **B** Densitometric quantification of Beclin1, LC3I/LC3II, p62, caspase-4, IL-1β and GSDMD in the heart and Becn1 and GSDMD in the spleen and liver. Data are expressed as mean ± SD (n = 3). ^*^P < 0.05 vs. WT group, ^**^P < 0.05 vs. WT-I/R group. **C** Beclin1, LC3, p62, caspase-4, IL-1β and GSDMD expression in WT and BECN1-Tg heart under Sham or 45 min ischemia/6 h of reperfusion was assessed by western blot analysis. **D** GSDMD expression in WT and BECN1-Tg spleen and liver after 45 min myocardial ischemia/6 h of reperfusion as assessed by western blot analysis. **E**, **F** Immunofluorescence staining and quantification of GSDMD in WT and BECN1-Tg heart in Sham or 45 min ischemia/6 h of reperfusion. Data are expressed as mean ± SD (n = 3). Scar bar = 20 μm. ^*^P < 0.05 vs. WT-I/R group. **G** Expression of GSDMD in WT and BECN1-Tg spleen and liver after 45 min myocardial ischemia/6 h of reperfusion as determined by immunofluorescence. **H** Quantification showed a remarkably decreased expression of GSDMD in BECN1-Tg group. Data are expressed as mean ± SD (n = 3). Scar bar = 20 μm. ^*^P < 0.05 vs. WT group

To examine whether up-regulation of Becn1 and autophagy levels influences caspase-4 activation mediated pyroptosis in mice. The levels of LC3, p62, caspase-4, IL-1β and GSDMD in WT and BECN1-Tg heart were assessed by western blotting. Firstly, Becn1 overexpression effect was observed, the expression of Becn1 was higher in the BECN1-Tg heart than that of in WT group (Fig. 5A, B, ** < 0.05). Our data also indicated that the basal levels of caspase-4 and IL-1β were significantly lower in BECN1-Tg group than WT group (Fig. 5B, C, ** < 0.05). Next, to further establish MI/R model and observe the changes of above proteins, the results showed that the levels of LC3-II/LC3I were significantly increased (Fig. 5B, C, ** < 0.05); while p62, caspase-4, IL-1β and GSDMD expression were significantly decreased in BECN1-Tg-I/R group compared to WT-I/R group (Fig. 5B, C, ** < 0.05). We also investigated expression of GSDMD in the spleen and liver after MI/R. The results were consistent with the heart tissue (Fig. 5D, B, ** < 0.05). Immunofluorescence analysis also indicated the expression of GSDMD was attenuated in BECN1-Tg-I/R group not only in the heart but also in the spleen and liver (Fig. 5E-H, ** < 0.05).

### Impaired autophagic flux promotes caspase-4 inflammasome activation in CMECs

**Fig. 6:**
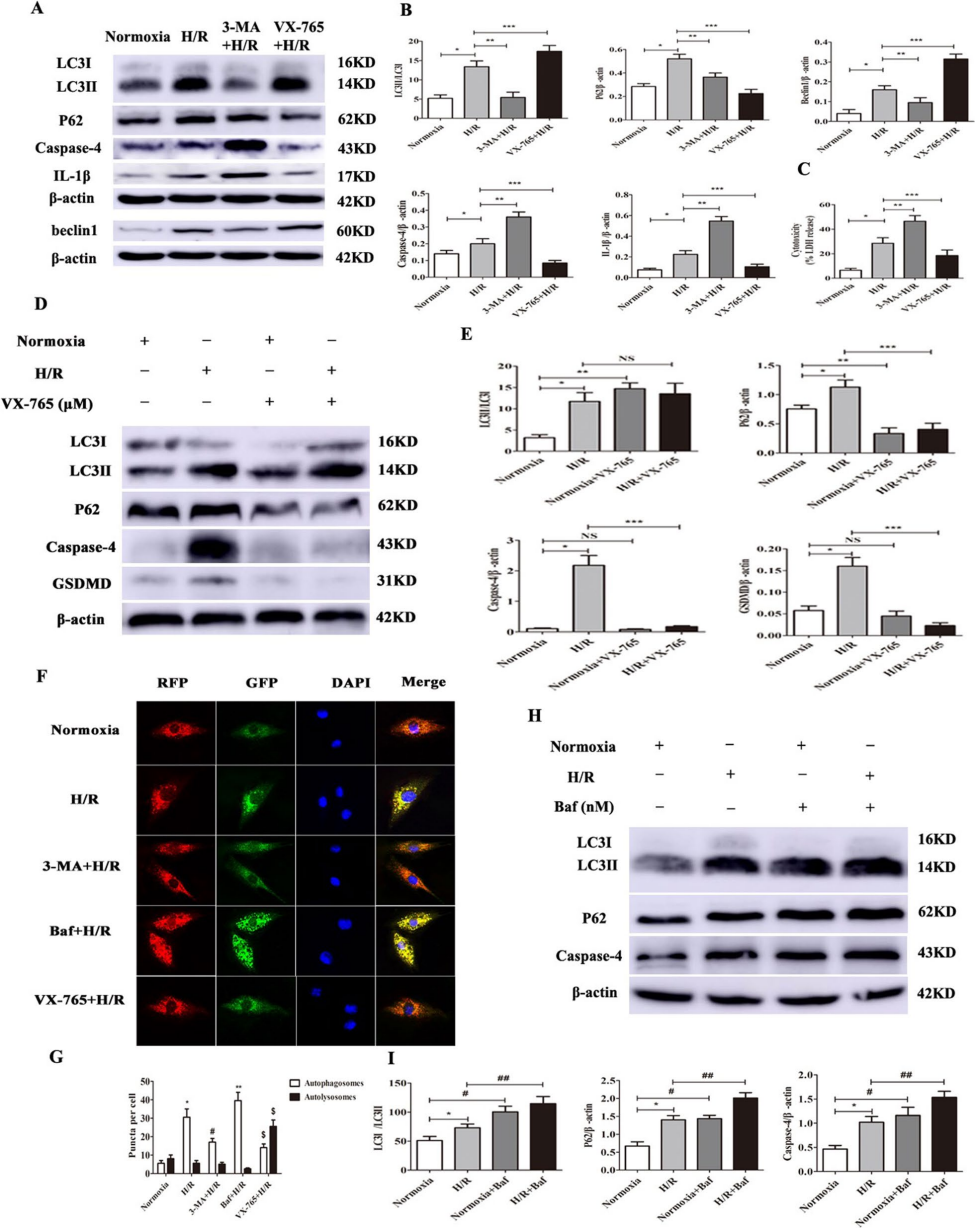
Inhibiting autophagic flux promotes caspase-4 inflammasome activation. **A**, **B** Expression and quantification of beclin1, LC3, p62, caspase-4 and IL-1β in CMECs cultured under normoxia or subjected to H/R (2/2 h) with or without 3-MA (5 mM), VX-765 (10 μM). Data are expressed as mean ± SD (n = 3). ^*^P < 0.05 vs. control group, ^**^P < 0.05 vs. 3-MA + H/R group, ^***^P < 0.05 vs. VX-765 + H/R group. **C** Analysis of LDH release in CMECs subjected to H/R (2/2 h) ± 3-MA (5 mM) or VX-765 (10 μM). Data are expressed as mean ± SD (n = 5). ^*^P < 0.05 vs. control group, ^**^P < 0.05 vs. 3-MA + H/R group, ^***^P < 0.05 vs. VX-765 + H/R group. **D**, **E** Expression and quantification of LC3, p62, caspase-4 and GSDMD in CMECs cultured under normoxia or subjected to H/R (2/2 h) with or without VX-765 (10 µM). Data are expressed as mean ± SD (n = 3). ^*^P < 0.05 vs. control group, ^**^P < 0.05 vs. control + VX-765 group, ^***^P < 0.05 vs. H/R + VX-765 group. **F** CMECs were cultured for 24 h after transfection with RFP-GFP-LC3. CMECs carring RFP-GFP-LC3 subjected to H/R were treated with or without 3-MA (5 mM), Baf (50 nM) or VX-765 (10 µM). Representative immunofluorescence images of CMECs expressing RFP-GFP-LC3; cell nuclei were stained with DAPI (blue). Red punta represent autophagosomes; yellow punta in merged picture represent autolysosomes. **G** Semi-quantitative analysis of autophagosomes and autolysosomes in each group. **H**, **I** Expression and quantification of LC3, p62 and caspase-4 in CMECs cultured under normoxia or subjected to H/R (2/2 h) with or without Baf (50 nM). Data are expressed as mean ± SD (n = 3). ^*^P < 0.05 vs. control group, ^#^*P* < 0.05 vs. control + Baf group, ^##^*P* < 0.05 vs. H/R + Baf group

To investigate the effect of autophagy on caspase-4 activation, CMECs were subjected to 2 h hypoxia followed 2 h re-oxygenation. In needed, H/R significantly increased caspase-4 activation as well as secretion of IL-1β (Fig. 6A, B, ** < 0.05); meanwhile the levels of beclin1, LC3-II/LC3I, p62 were also elevated (Fig. 6A, B, ** < 0.05). Furthermore, CMECs were stimulated with the autophagy inhibitor 3-MA for 2 h. The results indicated that following the treatment with 3-MA, the expression of beclin1, LC3-II/LC3I and p62 was decreased; whereas caspase-4 and IL-1β expression were further increased in CMECs (Fig. 6A, B, ** < 0.05). On the other hand, to explore the impact of caspase-4 on autophagy activity, caspase-4 inhibitor VX-765 was treated with CMECs for 2 h. The results showed that VX-756 pre-treatment suppressed caspase-4 activation, IL-1β secretion as well as pyroptosis; whereas VX-756 pre-treatment further increased the expression of beclin1 and LC3-II/LC3I and decreased p62 in CMECs (Fig. 6A, B, D, E, ** < 0.05). LDH release assay indicated H/R-induced cell death. In this connection, LDH release in CMECs were blocked by VX-765 and activated by 3-MA (Fig. 6C, ** < 0.05).

To monitor autophagic flux, given our findings above, the effect of autophagic flux of regulating the caspase-4 activation was further confirmed. Autophagic flux comprises induction, maturation and degradation of autophagosome. To monitor autophagic flux, RFP-GFP-LC3 was transfected into CMECs, using fluorescence microscope to detect the puncta. CMECs without H/R showed a basal level of autophagy as exhibited by a few autophagosomes and autolysosomes puncta. CMECs exposed to H/R or treatment Baf (inhibition lysosomal degradation) had massive autophagosomes and few autolysosome puncta. 3-MA treatment resulted in fewer autophagosomes and autolysosomes puncta. However, VX-765 treatment resulted in more autolysosomes and fewer autophagosomes (Fig. 6F, G, ** < 0.05). To confirm that caspase-4 activation regulated by autophagic flux during H/R, Baf was treated with CMECs. The result showed that Baf treatment significantly increased LC3II/LC3I and p62 expression; while caspase-4 expression was further increased (Fig. 6H, I, ** < 0.05).

### Becn1 overexpression driven autophagic flux inhibits caspase-4 inflammasome activation and pyroptosis in CMECs

**Fig. 7:**
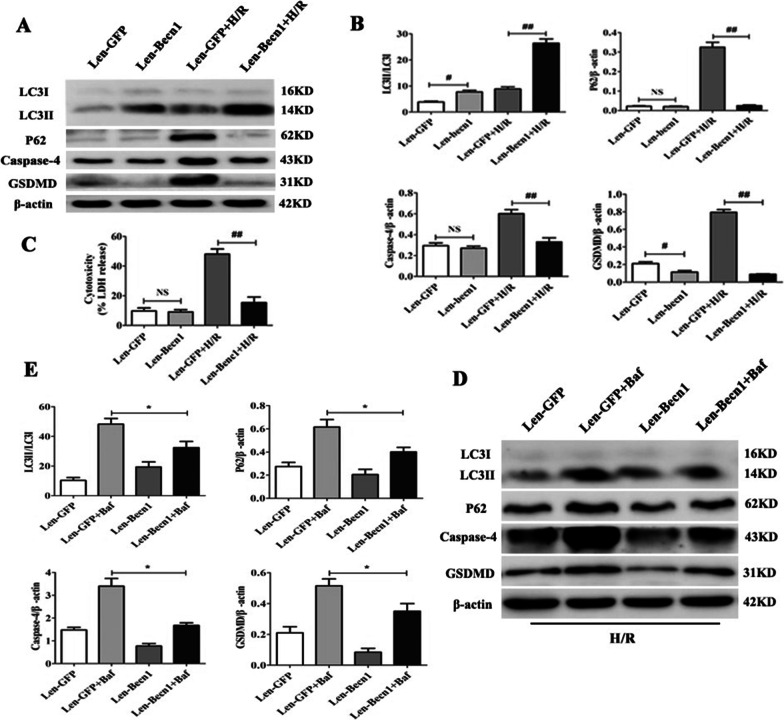
Beclin1 regulates autophagy flux through inhibiting caspase-4-mediated pyroptosis in CMECs. **A**, **B** Expression and quantification of LC3, P62, caspase-4 and GSDMD in CMECs cultured under normoxia or subjected to H/R (2/2 h). Data are expressed as mean ± SD (n = 3). ^*^P < 0.05 vs. control group, ^#^*P* < 0.05 vs. Len-GFP group, ^##^ *P* < 0.05 vs. Len-GFP + H/R group. **C** Analysis of LDH release in CMECs under normoxia or 2 h hypoxia/ 2 h of re-oxygenation. Data are expressed as mean ± SD (n = 5). ^##^*P* < 0.05 vs. Len-GFP + H/R group. **D**, **E** Expression and quantification of LC3, P62, caspase-4 and GSDMD in CMECs cultured under subjected to H/R (2/2 h). Data are expressed as mean ± SD (n = 3). ^*^P < 0.05 vs. Len-GFP + Baf group

Next, to determine the effect of up-regulation of beclin1 and autophagy on caspase-4 medicated pyroptosis, Len-Becn1 was transferred into CMECs. The levels of LC3, p62, caspase-4, and GSDMD in CMECs were assessed by western blot. The results indicated that Len-Becn1 group showed increased levels of LC3-II/LC3I compared to Len-GFP group (Fig. 7A, B, ** < 0.05), while p62 expression had no altered. However, LC3-II/LC3I expression was up-regulated, while p62 expression was decreased in Len-Becn1 group during H/R (Fig. 7A, B, ** < 0.05). Furthermore, caspase-4 activation and GSDMD expression in Len-Becn1 group was lower compared to that in Len-GFP group during H/R (Fig. 7A, B,* P* < 0.05). In addition, LDH Cytotoxicity Assay demonstrated that beclin1overexpression blocked LDH release in CMECs exposed to H/R (Fig. 7C, ** < 0.05). To further ensure beclin1 overexpression affects caspase-4-dependent pyroptosis during H/R. Cells was treated with Baf. The results showed accumulation of LC3 and P62 compared to in Len-GFP group (Fig. 7D, E, ** < 0.05). Nevertheless, transfection with Len-Becn1 in CMECs treated with Baf is able to restore autophagic flux compared to Len-GFP + Baf group (Fig. 7D, E, ** < 0.05).

## Discussion

**Fig. 8:**
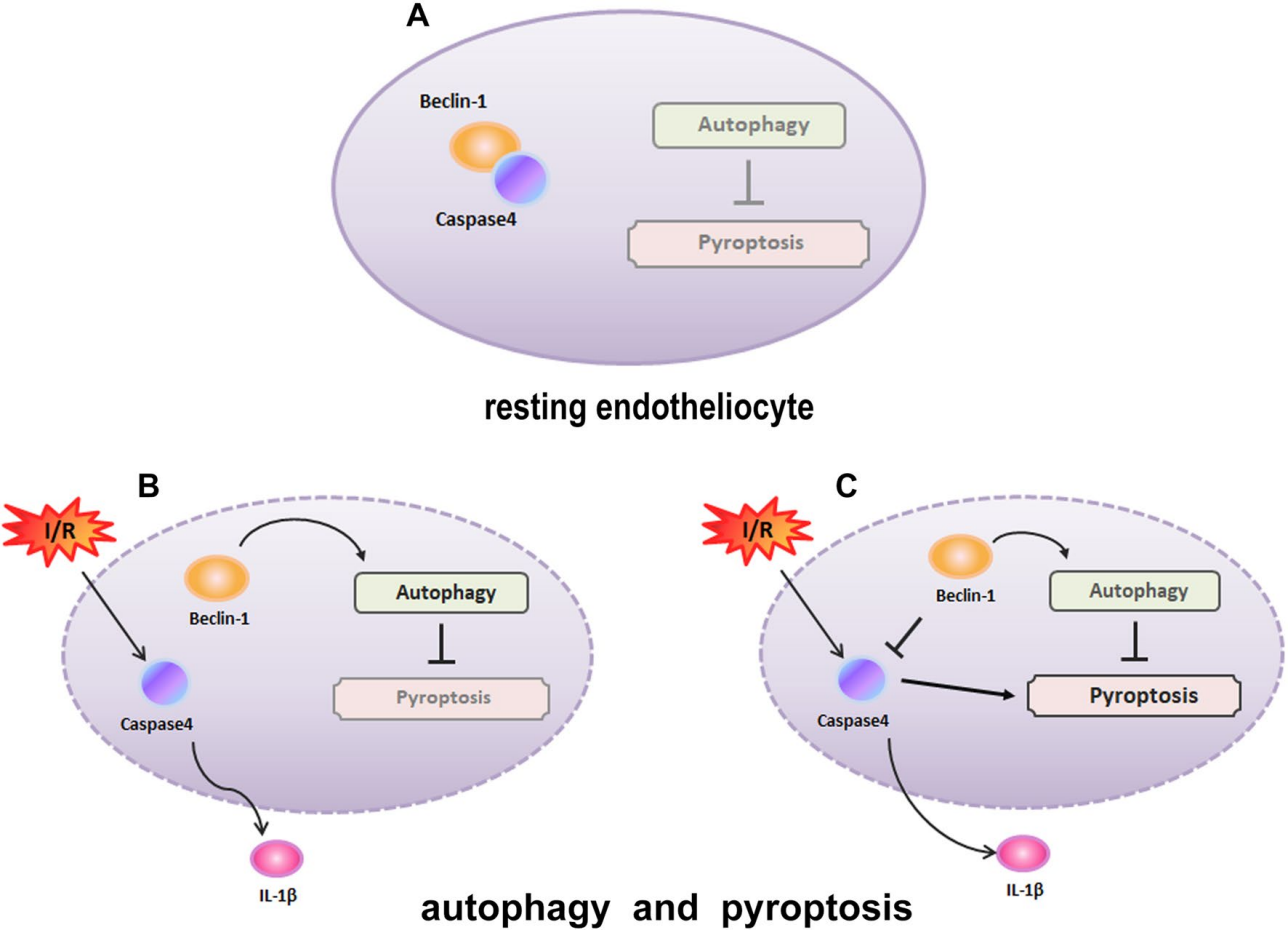
Autophagy regulates caspase-4 inflammasome and pyroptosis as cardiac microvascular endothelial cells (CMECs) responses to MI/R. **A** In resting CMECs, autophagy component beclin1 is complexed with caspase-4, inhibiting pyroptosis. **B**, **C** In response to I/R, caspase-4 inflammasome activation in CMECs, when caspase-4 inflammasome activation is exceeded, an increase in expression of beclin-1promotes autophagy activity to orchestrate caspase-4 activation and pyroptosis, which protects cells from I/R injury

This study identified the effects of increased Becn1 levels on caspase-4-dependment pyroptosis in CMECs in vitro after H/R and in vivo in a mouse I/R model. That Becn1-dependent autophagy regulated caspase-4 mediated pyroptosis was first reported in the mechanism of microvascular injury induced by MI/R. Of note, the up-regulation of Becn1 expression in mice and CMECs resulted in enhanced autophagic flux. We also provided evidence that caspase-4 inflammasome activation and pyroptosis were increased in both CMECs and the heart during MI/R. In addition, GSDMD cleaved by caspase-4 which promoted pyroptosis in the heart and CMECs was suppressed by beclin1 overexpression that promoted autophagic flux. More importantly, we have showed that beclin1 overexpression increased animal survival and attenuated microvascular injury and myocardial infarct size post-myocardial ischemia reperfusion (Fig. 8).

Previous studies have suggested that autophagy is recognized as a critical role in MI/R [24, 25]. However, generating beneficial or detrimental effect on IRI is still controversial. Our studies use transgenic mice with overexpression of Becn1 (*Becn1-Tg*) to test the role of Becn1-dependent autophagy in the heart during I/R. The current study showed that Becn1 overexpression promoted the signal of autophagy, alleviated microvascular damage, reduced infarct size and mitigated cardiac inflammation and cell death, and additionally, improved survival rate following I/R. These results indicate that Becn1-driven autophagy is a protective response in the heart during I/R.

However, how Becn1-dependent autophagy plays a protective role in response to I/R? Recently, numerous reports in the literature have indicated that autophagy plays a key role in regulation of inflmmasome [26, 27], but little is known about the function of Becn1 on noncanonical inflammasome caspase, CASP4, which mediated pyroptosis during MI/R. In the present study, we further explore whether caspase-4/GSDMD dependent pyroptosis plays a significant role in microvascular injury induced by I/R, and determine how Becn1-dependent autophagy regulates caspase-4-medicated pyroptosis and pro-inflammatory cytokines release during MI/R. We identified that caspase-4, GSDMD and IL-1β were up-regulated in both the heart and CMECs during MI/R, suggesting that caspase-4 mediated pyroptosis indeed plays a pivotal role in the pathophysiology of MI/R.

As unfettered pyroptosis can lead to myocardial tissue injury, targeting caspase-4-dependent pyroptosis may be a useful strategy for limiting IRI. Several studies have demonstrated that autophagy is a multifaceted modulator of cell death [28, 29]. But, at present, we do not fully understand how autophagy regulation of pyroptosis protects CMECs against IRI. Therefore, we hypothesized that Becn1 driven autophagy might inhibit pyroptosis by regulating caspse-4 inflammasome activation during MI/R. To support this hypothesis, we analyzed whether the up-regulation of Becn1 could suppress caspase-4 activation and pyroptosis in vivo in MI/R model established by using Becn1-Tg mice. Our results indicated in Becn1 overexpression I/R model not only LC3II/LC3I expression was increased but p62 expression was decreased. Along with this, caspase-4 activation, IL-1β levels and pyroptosis were decresed in BECN1-Tg I/R mice model. In addition, our observations in the spleen and liver were consistent with that in the heart, showing decreased pyroptosis after MI/R in BECN1-Tg mice. Taken together with our present results, it can be stated that Becn1 overexpression suppressed caspase-4-dependent pyroptosis induced by MI/R through enhancing autophagic flux.

To gain a deeper understanding of the potential mechanism of autophagy that regulates caspase-4 activation in vitro, molecular evidence of caspase-4 activation was investigated in CMECs. First, the effect of H/R on caspse-4 inflammasome activation was investigated. Our results indicated that caspase-4 activation and caspase-4 inflammasome-associated cytokines IL-1β were increased in CMECs exposed to H/R. This suggests that caspase-4 activation and pro-inflammatory cytokines IL-1β might be involve in microvascular endothelium injury mediated by H/R. Furthermore, how H/R deregulates autophagy signaling and thereby results in CMECs injury. Hence, we evaluate the expression changes of autophagic proteins Becn1, LC3II/ LC3I and p62, which were found to be up-regulated in CMECs during H/R. The results are consistent with previous study [30]. Our observations suggest that H/R may impair autophagic flux in CMECs.

To further determine H/R induced CMECs damage may be related to impaired autophagic flux. Subsequently, RFP-GFP-LC3 Lentivirus was transfected into CMECs to further monitor the effect of H/R on autophagic flux. Our observations showed that the incidence of autophagosomes was increased, but that of autolysosomes was decreased, indicating impairment of autophagolysosomal maturation in CMECs during H/R. This corroborates the results of western blot. The above results indicate that H/R induced caspase-4 inflammasome activation in CMECs that may be related to impaired autophagy. In order to confirm the effect of autophagy on caspase-4 inflammasome activation in CMECs, autophagy blocker 3-MA was used to treat CMECs before re-oxygenation. The results demonstrated that addition of 3-MA in CMECs had led to further activation of caspase-4 and resulted in increased expression of IL-1β; however, the expression levels of autophagic proteins beclin-1, LC3II/ LC3I and p62 were down-regulated. In CMECs transfected with RFP-GFP-LC3 lentivirus and treated with 3-MA, the numbers of both autophagosomes and autolysosomes were reduced. These data illustrate that inhibition of autophagy further promotes caspse-4 activation and IL-1β production. A recent study shed light on inhibition of inflammasome activation which promotes autophagy [31]. Arising from the above, we surmised that caspase-4 inhibitor VX-765 would enhance autophagy by impeding the activity of caspase-4 inflammasome. To support this hypothesis, caspase-4 inhibitor VX-765 was used to treat CMECs before re-oxygenation. Our data indicated that inhibition of caspase-4 increased autophagic protein beclin1 and LC3II/LC3I expression and decreased p62 expression in CMECs exposed to H/R. Meanwhile, VX-765 treat with CMECs transfected with RFP-GFP-LC3 lentivirus, the numbers of autophagosome were decreased while that of autolysosomes numbers were increased in CMECs. Our observations suggest that enhancing autophagic flux may protect CMECs against H/R injury mediated by caspase-4 inflammasome activation.

Previous experimental studies have highlighted the cross-talk between autophagy and inflammasome activation, the cellular machinery is related to the elimination of cellular components and maintenance of intracellular homeostasis [26, 32]. The present results demonstrated that beclin1 could suppress caspase-4 mediated pyroptosis by enhancing autophagic flux in Becn1-Tg mice. Inhibition of caspase-4 activation conversely promoted the expression of Becn1 in vitro. Based on these data, we further explored whether increased Becn1 levels would have an effect on caspas-4-dependment pyroptosis in CMECs during H/R. For this, lentivirus carrying Becn1 gene (Len-Becn1) and lentiviral vector carrying green fluorescence (Len-GFP) were respectively transfected into CMECs subjected to H/R. Caspase-4 activation and pyroptosis were examined in CMECs transfected with Len-GFP during H/R. The results support that CMECs may undergo caspase-4-dependent pyroptosis induced by H/R. However, in vitro genetic enhancement in Becn1 resulted in increased expression of LC3II/LC3I, but the expression of p62 was not altered, while pyroptosis was decreased in CMECs without exposure to H/R. Furthermore, the effect of up-regulation of beclin1 on caspase-4-dependent pyroptosis in CMECs exposed to H/R was observed, These data demonstrated that genetic enhancement in beclin1 resulted in increased expression of LC3II/LC3I and decreased p62 expression, while caspase-4 activation and pyroptosis were markedly suppressed, which is in agreement with the results in vivo. These findings demonstrate that Becn1-driven autophagy suppressed caspase-4-mediated pyroptosis, which may protect microvascular endothelial cell against H/R injury.

In summary, our data have provided the molecular mechanism whereby Becn1-driven autophagy may regulate caspase-4-mediated pyroptosis. This would be helpful to determine the potential molecular biomarker and therapeutic targeting of autophagic process for ameliorration myocardial reperfusion-induced microvascular injury.

### Limitations of study

Our study has demonstrated that Becn1-driven autophagy suppressed caspase-4-mediated pyroptosis, which may protect microvasuclar dysfunction against MI/R. IRI may be a key pointcut in the process of cell death. However, the forms of cell death include necroptosis, apoptosis and pyroptosis, which was occur in MI/R. Moreover, the closely relationship among apoptosis, necroptosis, and pyroptosis remains unknown in the process of microvascular injury following myocardial reperfusion. Previous studies indicated that Gasdermin was specifically cleaved by caspase-3, which mediated apoptosis to pyroptosis [33]; Mitochondrial permeability transition pore activates caspase-4/11-caspase-3-GSDME signal axis to secondary pyroptotic cell death [34]. Based on the data, the interactions between the forms of cell death are very complicated. Therefore, it is very important to clarify the molecular events to mediate apoptosis, necroptosis, and pyroptosis in response to MI/R in the near future. In addition, whether pyroptosis mediated by ischemia–reperfusion through other regulatory pathways is also a question worth exploring in the near future.

Potential translation to humans in the study is that Becn1 may act as an important intervention tool or potential drug for the patients with microvascular dysfunction post-myocardial ischemia reperfusion in the furture.

## Data Availability

The datasets supporting the conclusions of this article are included within the article files as well as materials prepared are available from the corresponding author on reasonable request.

## Abbreviations

NLRP3: NOD-like receptor protein 3
Caspase-1: Cysteinyl aspartate-specific proteinase-1
IL-1β: Interleukin 1β
3-MA: 3-Mthyladenine
CMECs: Cardiac microvascular endothelial cells
LC3: Microtubule-associated protein light chain 3
LDH: Lactate dehydrogenase
CK: Creatine kinase
TTC: Tetracycline
WT: Wild type
Becn1-Tg: Transgenic mice with overexpression of Beclin 1
